# Elranatamab in Relapsed/Refractory Multiple Myeloma: A Multicenter Real-World Study from Türkiye

**DOI:** 10.3390/jcm15166232

**Published:** 2026-08-12

**Authors:** Aslı Bozdemir, Sibel Hacıoğlu, Gülsüm Akgün Çağlıyan, Nevin Alayvaz Aslan, Süleyman Utku Uzun, Kayıhan Kara, Utku Iltar, Orhan Kemal Yücel, Ünal Ataş, Selin Arslan Kirezli, Ali İhsan Gemici, İnci Alacacıoğlu, Mustafa Kemal Yeniay, Oktay Bilgir, Zehra Narlı Özdemir, Handan Haydaroğlu Şahin, Ayşe Uysal, Zekeriya Aksöz, Zeynep Tuğba Güven, Kemal Aygün, Atakan Tekinalp, Mehmet Yılmaz, Cansu Atmaca Mutlu, Gökhan Pektaş, Ozan Salim, Nil Güler

**Affiliations:** 1 Division of Hematology, Pamukkale University Hospital, Denizli 20070, Türkiye; skabukcu@gmail.com (S.H.); drgulsumakgun@gmail.com (G.A.Ç.); nevinalayvaz@gmail.com (N.A.A.); kayihankara@gmail.com (K.K.); nilvecay@yahoo.com (N.G.); 2 Division of Epidemiology, Pamukkale University Hospital, Denizli 20070, Türkiye; utkuuzun402@gmail.com; 3 Division of Hematology, Akdeniz University Hospital, Antalya 07070, Türkiye; utkuiltar@akdeniz.edu.tr (U.I.); orhankemalyucel@akdeniz.edu.tr (O.K.Y.); unalatas@akdeniz.edu.tr (Ü.A.); selinkirezli@akdeniz.edu.tr (S.A.K.); ozansalim@akdeniz.edu.tr (O.S.); 4 Division of Hematology, Dokuz Eylül University Hospital, İzmir 35340, Türkiye; ali.gemici@deu.edu.tr (A.İ.G.); inci.alacacioglu@deu.edu.tr (İ.A.); 5 Division of Hematology, İzmir City Hospital, İzmir 35530, Türkiye; mustafakemalyeniay@gmail.com (M.K.Y.); obilgir@hotmail.com (O.B.); 6 Division of Hematology, TOBB ETÜ Faculty of Medicine Hospital, Ankara 06560, Türkiye; zehranarli@hotmail.com; 7 Division of Hematology, Gaziantep University Hospital, Gaziantep 27310, Türkiye; handansahin@gantep.edu.tr; 8 Division of Hematology, Fırat University Hospital, Elazığ 23119, Türkiye; drayseorucuysal@gmail.com (A.U.); zaksoz@firat.edu.tr (Z.A.); 9 Division of Hematology, Adana City Hospital, Adana 01370, Türkiye; zeynepguven@erciyes.edu.tr; 10 Division of Hematology, İzmir Atatürk Training and Research Hospital, İzmir 35360, Türkiye; drkml_aygun@hotmail.com; 11 Division of Hematology, Necmettin Erbakan University Faculty of Medicine Hospital, Konya 42080, Türkiye; atekinalp@erbakan.edu.tr; 12 Division of Hematology, Sanko University Hospital, Gaziantep 27090, Türkiye; mmyilmaz246@hotmail.com; 13 Division of Hematology, Buca Seyfi Demirsoy Training and Research Hospital, İzmir 35390, Türkiye; cansu_atmaca@yahoo.com; 14 Division of Hematology, Muğla Training and Research Hospital, Muğla 48000, Türkiye; gokhanpektas@mu.edu.tr

**Keywords:** elranatamab, bispecific antibody, multiple myeloma, real-world data, infectious complications

## Abstract

**Background:** Elranatamab, a bispecific antibody targeting BCMA and CD3, has demonstrated clinical activity in relapsed/refractory multiple myeloma. Real-world evidence regarding infectious complications and supportive care remains limited. We evaluated the early clinical activity, safety profile, infectious complications, and supportive care practices associated with relapsed/refractory multiple myeloma (RRMM). This study represents one of the first multicenter real-world evaluations of elranatamab in Türkiye. **Methods:** This multicenter retrospective study included 87 patients with relapsed/refractory multiple myeloma treated with elranatamab. Clinical characteristics, treatment responses, immune-mediated toxicities, infectious complications, and supportive care practices were assessed. Overall response rate (ORR), progression-free survival (PFS), and overall survival (OS) were evaluated. Multivariable analyses were performed to identify factors associated with treatment response and clinical outcomes. **Results:** At 3 months, ORR was 47.1% in the ITT population, 54.7% in the mITT population, and 83.7% among evaluable patients; corresponding 6-month ORRs were 31.0%, 42.2%, and 81.8%, respectively. The high proportion of patients without landmark response assessments primarily reflected insufficient follow-up, early death, or disease progression. Elevated LDH remained independently associated with lower response probability and inferior clinical outcomes. Cytokine release syndrome (CRS) occurred in 69% of patients, with grade ≥ 3 events in 5.7%, whereas immune effector cell-associated neurotoxicity syndrome (ICANS) was infrequent (4.6%) and no grade ≥ 3 events occurred. Grade ≥ 3 infections occurred in 39% of patients, including CMV events requiring antiviral treatment in 24.1%. No HBV reactivation occurred among patients receiving antiviral prophylaxis. Median PFS and OS were 8.1 and 10.6 months, respectively. **Conclusions:** Elranatamab demonstrated early clinical activity and a manageable safety profile in a heavily pretreated real-world RRMM population. Infectious complications, including CMV events, remained clinically relevant, emphasizing the importance of supportive care. Longer follow-up is needed to characterize long-term outcomes.

## 1. Introduction

Multiple myeloma (MM) continues to be an incurable hematologic cancer, notwithstanding significant progress in treatment techniques in recent years [1]. Elranatamab, a humanized bispecific antibody, simultaneously targets the CD3 receptor on T lymphocytes and the B-cell maturation antigen (BCMA) expressed on multiple myeloma cells. By redirecting T cells toward myeloma cells, it induces immune-mediated cytotoxicity. Elranatamab has received approval as a monotherapy for the treatment of relapsed/refractory multiple myeloma [2].

The efficacy and safety of elranatamab in patients with RRMM were demonstrated in the phase II MagnetisMM-3 trial; however, patients enrolled in clinical trials generally represent selected populations meeting predefined eligibility criteria [3]. Although real-world data on elranatamab have increasingly emerged following its approval, currently available evidence remains limited [4].

In particular, data regarding clinical outcomes in heavily pretreated patients, those with penta-refractory disease, extramedullary involvement, or significant comorbidities remain scarce. Furthermore, evaluation of real-world data is essential to better characterize infections and treatment-related toxicities associated with elranatamab therapy.

In this multicenter real-world study, we aimed to evaluate the efficacy, safety profile, prognostic factors, infectious complications, and supportive care approaches in patients with RRMM treated with elranatamab, while also examining short-term clinical outcomes.

The study was designed to extend the available real-world evidence by evaluating a heavily pretreated population with a high burden of penta-class refractory disease and extramedullary involvement, together with a detailed assessment of infectious complications and potential prognostic factors.

## 2. Methods

### 2.1. Study Design and Patients

The research involved 12 different hospitals in Türkiye and was a retrospective cohort study. Eligible for inclusion were all adult patients (≥18 years) with RRMM who received at least one dose of elranatamab between January 2025 and January 2026. In Türkiye, patients with RRMM were considered eligible for elranatamab treatment only if they had previously received at least four lines of therapy, including immunomodulatory agents, proteasome inhibitors, and anti-CD38 monoclonal antibodies, and had experienced disease progression during their most recent treatment. To better reflect routine clinical practice, no additional exclusion criteria beyond eligibility for elranatamab treatment were applied. Clinical, laboratory, and treatment-related data were extracted from institutional electronic medical records by site investigators using a standardized case report form.

The study was conducted in accordance with the Declaration of Helsinki and was approved by the relevant institutional review boards of the participating centers.

### 2.2. Definitions and Endpoints

At the 3- and 6-month mark, the disease’s response was evaluated using criteria established by the International Myeloma Working Group (IMWG). The percentage of patients who achieved a partial response or better (≥PR) at the corresponding time point was used to define the overall response rates (ORR) at months 3 and 6. Stringent complete response (sCR) was defined according to IMWG criteria; however, as all required components were not routinely available across centers and time points, reported sCR rates should be interpreted with caution [5].

Cytokine release syndrome (CRS) and immune effector cell-associated neurotoxicity syndrome (ICANS) were evaluated according to the consensus criteria of the American Society for Transplantation and Cellular Therapy (ASTCT). ICANS was assessed through clinical evaluation at each participating center [6].

According to the Common Terminology Criteria for Adverse Events (CTCAE) version 5.0, the most severe hematologic adverse events that occurred within 90 days of treatment beginning were graded. These represent worst-grade cytopenias within the 90-day window and do not distinguish treatment-emergent events from pre-existing baseline cytopenias [7].

A CMV event was operationally defined as a detected CMV event for which the treating physician initiated CMV-directed antiviral therapy. CMV PCR surveillance and treatment thresholds were determined according to local institutional practice. Surveillance strategies were categorized as no scheduled CMV PCR surveillance, testing only upon clinical suspicion, monthly surveillance, or surveillance every 2 weeks or more frequently. Symptomatic status, quantitative treatment thresholds, recurrence, and CMV-attributable deaths or treatment discontinuations were not systematically captured.

HBV reactivation events were identified from medical records based on virologic and/or serologic evidence documented by treating physicians and were assessed according to local institutional practice at participating centers.

High-risk cytogenetics were characterized by the presence of del(17p), t(4;14), t(14;16), or 1q gain/amplification [8]. Refractoriness to a minimum of three drugs—an immunomodulatory drug (IMiD), an anti-CD38 monoclonal antibody, and a proteasome inhibitor (PI)—was considered a triple-class refractory condition. Refractoriness to a minimum of two PIs, two IMiDs, and one anti-CD38 monoclonal antibody was considered to constitute pentaclass refractory status [9].

The primary endpoint was ORR at 3 and 6 months, assessed using ITT, mITT, and evaluable-patient analyses. In the ITT analysis, all non-evaluable patients, including those who died, experienced disease progression, had insufficient follow-up, or had missing response assessments before the respective landmark, were considered non-responders. In the mITT analysis, only patients with insufficient follow-up who had not yet reached the respective landmark were excluded from the denominator, while those who died, progressed, or had missing response assessments remained classified as non-responders. The evaluable-patient analysis included only patients with a documented response assessment at the respective landmark. Secondary endpoints included safety, infection outcomes, OS, and PFS. All patients were censored at the last follow-up if they did not have any events; OS and PFS were calculated from the first dose of elranatamab.

### 2.3. Statistical Analysis

Descriptive data were presented as median with interquartile range (IQR) for continuous variables and as frequency with percentage for categorical variables. The Kaplan–Meier method was used for survival analysis, and the log-rank test was used to compare groups. We computed the median follow-up using the reverse Kaplan–Meier approach.

In univariate analyses, logistic regression was used for ORR, whereas Cox proportional hazards regression was used for PFS and OS, and odds ratios (OR) and hazard ratios (HR) were reported with 95% confidence intervals (CI). The candidate variables included in the analysis were age, sex, ECOG performance status, ISS/R-ISS stage, extramedullary disease (EMD), high-risk cytogenetics, penta-class refractoriness, line of therapy (LOT), prior autologous stem cell transplantation, bone marrow plasma cell infiltration, LDH level, renal function, baseline hypogammaglobulinemia, and prophylactic IVIg use.

Given the limited number of OS (*n* = 29) and PFS (*n* = 37) events, multivariable Cox models were restricted to three covariates to minimize overfitting, based on an approximate 10-events-per-variable approach. ECOG performance status, LOT, and LDH were selected as clinically relevant covariates representing patient functional status, prior treatment burden, and disease burden, respectively. EMD was additionally evaluated as a fourth candidate variable using the likelihood ratio test but was not retained in the final survival models because it did not improve model fit. Multivariable logistic regression was also performed for ORR, with EMD additionally evaluated as an exploratory variable for 3-month ORR.

Missing data were not imputed. Complete-case analysis was performed for each endpoint, and the sample size for each analysis was reported accordingly. The proportional hazards assumption was evaluated using Schoenfeld residuals. Response rates were reported with exact 95% confidence intervals. A two-tailed *p*-value of <0.05 was considered statistically significant. R version 4.5.2 (R Foundation for Statistical Computing, Vienna, Austria) was used for all analyses.

## 3. Results

### 3.1. Patient Characteristics

From January 2025 to January 2026, 87 patients across 12 hospitals in Türkiye received at least one dose of elranatamab and were included in the analysis. The baseline characteristics are presented in Table 1.

The median age was 63 years (IQR 57–69). ECOG performance status (PS) was 0–1 in 51 patients (58.6%) and ≥2 in 36 patients (41.4%). ISS stage I, II, and III disease was observed in 21.8%, 26.4%, and 51.7% of patients, respectively. Among evaluable patients, R-ISS stage I, II, and III distribution was 11.8%, 44.7%, and 43.4%, respectively. High-risk cytogenetic abnormalities were identified in 21.9% of patients with available cytogenetic data, and extramedullary disease (EMD) was documented in 59.8%.

The cohort consisted of heavily pretreated patients; 98.9% were triple-class refractory, while 73.6% were penta-class refractory. Elranatamab was initiated at a median line of therapy (LOT) of 5 (IQR 5–6), and 86.2% of patients had previously undergone autologous stem cell transplantation.

### 3.2. Response

Response distributions are summarized in Table 2. Response was evaluable in 49 patients (56.3%) at 3 months and 33 patients (37.9%) at 6 months.

Among the 38 non-evaluable patients at 3 months, 13 died before response assessment, 5 experienced disease progression, 12 had insufficient follow-up (<3 months), and 8 had missing assessment data. At 6 months, the 54 non-evaluable patients included 21 deaths before response assessment, 7 progressions, 23 patients with insufficient follow-up (<6 months), and 3 with missing assessment data.

At 3 months, ORR was 47.1% in the ITT population, 54.7% in the mITT population, and 83.7% among evaluable patients (41/49; 95% CI 70.3–92.7%). Corresponding 6-month ORRs were 31.0%, 42.2%, and 81.8% (27/33; 95% CI 64.5–93.0%), respectively. Among evaluable patients, ≥VGPR and ≥CR rates were 57.1% and 30.6% at 3 months and 78.8% and 63.6% at 6 months, respectively. No sCR was documented at either timepoint.

Among 30 patients evaluable at both timepoints, response deepening, stable response, and response deterioration were observed in 33.3%, 46.7%, and 20.0% of patients, respectively.

### 3.3. Survival

At a median follow-up of 6.2 months (95% CI 4.6–7.3), 29 deaths (33.3%) and 37 PFS events (42.5%) had occurred. Median OS and PFS were 10.6 months (95% CI 7.6–NR) and 8.1 months (95% CI 5.7–NR), respectively. Given the limited follow-up duration, these median survival estimates should be interpreted with caution. The 3-, 6-, and 9-month OS rates were 75.9%, 70.2%, and 53.0%, respectively, while the corresponding PFS rates were 70.3%, 60.0%, and 43.2%. Numbers at risk and fixed-time Kaplan–Meier estimates are provided in Supplementary Table S1.

In univariate analyses, ECOG PS ≥ 2, LDH > 300 U/L, eGFR ≥ 60 mL/min, and prior SCT were significantly associated with both PFS and OS (Table 3).

In multivariable analyses, elevated LDH was associated with inferior outcomes across response and survival endpoints. LDH > 300 U/L was associated with lower 3-month ORR (aOR 0.21, 95% CI 0.05–0.69, *p* = 0.015), while each 100 U/L increase in LDH was associated with shorter PFS (aHR 1.75, 95% CI 1.36–2.25, *p* < 0.001) and OS (aHR 1.94, 95% CI 1.50–2.51, *p* < 0.001). ECOG PS and LOT were not independently significant in multivariable models (Table 4). The proportional hazards assumption was satisfied for the primary multivariable Cox models (global Schoenfeld *p* = 0.34 for PFS and *p* = 0.49 for OS).

### 3.4. Safety and Infections

CRS occurred in 60 patients (69.0%), most of whom experienced grade 1–2 events; grade ≥ 3 CRS occurred in 5 patients (5.7%). Median onset was day 1 (IQR 1–2), and tocilizumab was administered in 22 patients (25.3%). ICANS occurred in 4 patients (4.6%), all grade 1–2. Within 90 days of treatment initiation, grade ≥ 3 lymphopenia, thrombocytopenia, and neutropenia occurred in 65 (74.7%), 34 (39.1%), and 21 (24.1%) patients, respectively. Tumor lysis syndrome occurred in 1 patient (1.1%). Treatment interruption and permanent discontinuation occurred in 28 (32.2%) and 22 (25.3%) patients, respectively (Table 5).

Any infection occurred in 52 patients (59.8%), including grade ≥ 3 infections in 34 (39.1%). Pneumonia was the predominant severe infection, but febrile neutropenia and sepsis were observed in 22 (25.3%) and 16 (18.4%) individuals, respectively. Antiviral-requiring CMV events occurred in 21 patients (24.1%). No scheduled CMV PCR surveillance was used in 19 patients (21.8%), testing was performed only upon clinical suspicion in 27 (31.0%), monthly surveillance was performed in 2 (2.3%), and surveillance every 2 weeks or more frequently was performed in 39 (44.8%). CMV events were detected in 1/19 (5.3%), 6/27 (22.2%), 0/2 (0%), and 14/39 (35.9%) patients in these respective groups. Among the 21 patients with CMV events, ganciclovir was used in 6 (28.6%) and valganciclovir in 17 (81.0%); none received foscarnet. Antiviral treatment categories were not mutually exclusive. (Supplementary Table S3).

HBsAg positivity was documented in 4 patients and anti-HBc positivity in 18, including all 4 HBsAg-positive patients. All 18 anti-HBc-positive patients (4 HBsAg-positive and 14 HBsAg-negative) received antiviral prophylaxis, and no HBV reactivation was observed during a median follow-up of 6.3 months, estimated by the reverse Kaplan–Meier method (Table 6). Prophylactic IVIg was administered to 68 patients (78.2%). An exploratory, unadjusted comparison of infection outcomes according to IVIg use is provided in Supplementary Table S2.

## 4. Discussion

This multicenter real-world study assessed the efficacy, safety profile, infectious complications, supportive care practices, and survival outcomes related to elranatamab in patients with relapsed/refractory multiple myeloma (RRMM). Given the high rates of penta-class refractory disease and extramedullary involvement, our cohort represented a clinically challenging and heavily pretreated patient population. Beyond providing additional real-world efficacy and safety data, our study extends the existing evidence through the detailed assessment of infectious complications and the evaluation of baseline LDH as a potential prognostic marker.

In our study, elranatamab demonstrated meaningful clinical activity despite the high prevalence of adverse disease features, including penta-class refractory disease and extramedullary involvement. At 3 months, ORR was 47.1% in the ITT population, 54.7% in the mITT population, and 83.7% among evaluable patients; corresponding 6-month ORRs were 31.0%, 42.2%, and 81.8%, respectively. Given the variable follow-up duration in routine clinical practice and the substantial proportion of patients without landmark response assessments, primarily due to insufficient follow-up, early death, or disease progression, these complementary estimates should be interpreted together. The lower response rates in the ITT analysis reflect the inclusion of non-evaluable patients as non-responders, whereas rates based only on evaluable patients may overestimate treatment response. The relatively lower mITT response rates compared with the ORR reported in the MagnetisMM-3 study were likely influenced by limited follow-up duration and the inability of some patients to reach landmark response assessments, particularly at later time points [3].

In the French compassionate-use program, elranatamab also demonstrated clinically meaningful activity despite a heavily pretreated and clinically challenging patient population, although response rates were numerically lower than those observed in our cohort [2].

In the subset of patients with available PET-CT assessment, high metabolic response rates, particularly at 3 months, further supported the clinical activity of elranatamab in a real-world setting. However, PET-CT was performed according to clinician discretion and not routinely in all patients. In addition, the 3- and 6-month PET-CT assessments represented overlapping but non-identical patient subsets, precluding direct longitudinal comparison between the two timepoints.

Elevated LDH remained significantly associated with inferior outcomes in the adjusted models, including a lower probability of achieving a 3-month response (aOR 0.21, 95% CI 0.05–0.69, *p* = 0.015), shorter PFS (aHR 1.75 per 100 U/L increase, 95% CI 1.36–2.25, *p* < 0.001), and shorter OS (aHR 1.94 per 100 U/L increase, 95% CI 1.50–2.51, *p* < 0.001). A recent real-world study similarly identified elevated LDH as an independent prognostic marker associated with inferior PFS (aHR 1.27, *p* < 0.001) and OS (aHR 1.36, *p* < 0.001) [10].

The persistence of this association when LDH was analyzed as a continuous variable further supports the relationship between increasing LDH levels and poorer outcomes. However, given the limited number of events and the parsimonious multivariable models, these findings should be interpreted cautiously and require validation in larger cohorts. Given the retrospective, single-arm design of our study, these findings do not establish LDH as a predictive biomarker of elranatamab efficacy. Elevated LDH may instead reflect greater disease burden or unfavorable disease biology and should therefore be considered a potential prognostic marker.

In our study, immune-mediated toxicities associated with elranatamab were generally observed to be manageable and tolerable. CRS was observed in 69% of patients, whereas grade ≥ 3 events occurred in only 5.7%. ICANS was rare, occurring in 4.6% of patients, and no grade ≥ 3 events were recorded. In the MagnetisMM-3 study, CRS and ICANS were reported in 56.3% and 3.4% of patients, respectively, with similarly low rates of severe immune-mediated toxicities [3]. Overall, our findings support the manageable safety profile of elranatamab in routine clinical practice.

Similar patterns of predominantly low-grade CRS and infrequent ICANS have been reported with other bispecific antibodies used in RRMM, suggesting that the immune-mediated toxicities observed in our cohort are not unique to elranatamab and are generally consistent with the broader experience reported for this treatment approach [11,12].

Infections were frequent in our study, with grade ≥ 3 infections observed in 39% of patients. Pneumonia rates were similar to those reported in MagnetisMM-3, whereas sepsis was more common in our cohort (18.4% vs. 5.7%) [3]. This may be related to the heavily pretreated nature of our patient population, particularly the high proportion of penta-class refractory disease.

Similar infectious concerns have also been reported with other bispecific antibodies in multiple myeloma, particularly with BCMA-directed agents, and infection risk is increasingly recognized as an important issue with this treatment class [13,14].

BCMA-directed bispecific antibody therapy may increase susceptibility to infections through several mechanisms. Depletion of normal plasma cells and other B-cell compartments may impair humoral immunity. Treatment-related hypogammaglobulinemia may further increase this risk. Lymphopenia and sustained T-cell engagement may also contribute to impaired immune responses. These changes may increase susceptibility to bacterial and opportunistic viral infections. Immunoglobulin replacement and antiviral prophylaxis may therefore play an important role in supportive care. Recent evidence suggests that immunoglobulin replacement may reduce the risk of severe infections. However, the optimal timing, dose, and duration of IVIg remain uncertain [15,16].

CMV events requiring antiviral therapy may be an important complication during elranatamab treatment. In our study, antiviral-requiring CMV events were observed in 24.1% of patients. Event detection was more frequent among patients undergoing scheduled PCR surveillance than among those tested only upon clinical suspicion or without scheduled surveillance; however, surveillance and treatment approaches varied across centers. This pattern is therefore susceptible to ascertainment bias and should not be interpreted as evidence that more intensive monitoring increases CMV risk. Our findings are in line with emerging real-world data reporting clinically relevant CMV events requiring antiviral therapy during elranatamab treatment, including recurrent reactivation despite antiviral therapy and prolonged immune dysfunction [17,18]. These findings support consideration of CMV monitoring during elranatamab treatment, particularly in heavily pretreated patients, while the optimal surveillance schedule and treatment threshold remain uncertain.

Although data regarding HBV reactivation associated with elranatamab remain limited, no HBV reactivation was observed among the 18 anti-HBc-positive patients who received antiviral prophylaxis. However, HBV DNA monitoring was performed according to local institutional practice and was not standardized across participating centers, while detailed monitoring intervals and specific antiviral prophylaxis regimens were not systematically captured. Given the small number of patients and these limitations, no firm conclusions regarding HBV reactivation risk or the effectiveness of antiviral prophylaxis can be drawn.

The median PFS and OS were 8.1 months and 10.6 months, respectively. However, given the relatively short follow-up duration in our cohort, survival outcomes remain immature, and direct comparisons with long-term follow-up data from MagnetisMM-3 should be interpreted cautiously [3].

Several limitations must be considered in interpreting the results of this study. Due to the retrospective design, some clinical data were missing or not systematically recorded, which may have influenced the results. The relatively short median follow-up duration, reflecting the recent introduction of commercial elranatamab in Türkiye, limits the maturity of survival outcomes and represents an inherent limitation of early real-world experience. Longer-term follow-up is required to more accurately assess long-term efficacy and survival outcomes. In addition, the absence of response data for all patients at the predefined 3- and 6-month landmark time points, as well as the fact that best overall response was not systematically captured, limits the interpretation of response. Finally, infection monitoring and supportive care practices, particularly for viral infections (e.g., CMV and HBV), were not standardized across participating centers. CMV symptoms, quantitative treatment thresholds, recurrence, and infection-attributable deaths or treatment discontinuations were not systematically captured. Detailed information regarding IVIg initiation, dosing, duration, and specific indications, as well as patient-level data on antiviral, antibacterial/PJP prophylaxis, G-CSF use, and vaccination practices, was not systematically captured.

Reasons for treatment interruption and permanent discontinuation, as well as treatment exposure duration, were not systematically captured across participating centers.

Despite these limitations, the multicenter real-world design and detailed evaluation of infectious complications provide useful insights into the use of elranatamab in heavily pretreated RRMM patients. Additional investigations involving larger cohorts and extended observation periods are warranted to clarify the most appropriate approaches for infection monitoring and supportive care during elranatamab therapy.

## 5. Conclusions

Elranatamab demonstrated meaningful clinical activity in a heavily pretreated real-world RRMM population. Immune-mediated toxicities, including CRS and ICANS, were generally manageable, although infectious complications, particularly viral infections, remained clinically relevant. Elevated baseline LDH was associated with poorer outcomes and may have potential prognostic significance rather than specifically predicting elranatamab efficacy. Our findings provide early real-world evidence of the use of elranatamab in routine clinical practice. Given the substantial burden of infectious complications observed in this cohort, further efforts are needed to standardize supportive-care strategies and to evaluate their impact prospectively. Future investigations involving broader real-world cohorts and extended observation periods may help clarify long-term outcomes and refine supportive care approaches.

## Figures and Tables

**Table 1:** Baseline Patient Characteristics (*n* = 87).

Characteristic	Value
**Age, years**—median (IQR; range)	63 (57–69; 46–81)
Age	
<75 years—*n* (%)	83 (95.4%)
≥75 years—*n* (%)	4 (4.6%)
**Sex**	
Female, *n* (%)	44 (50.6%)
Male, *n* (%)	43 (49.4%)
**ECOG performance status**—*n* (%)	
0–1	51 (58.6%)
2	28 (32.2%)
3–4	8 (9.2%)
**Heavy chain type**—*n* (%)	
IgG	63 (72.4%)
IgA	23 (26.4%)
Non-secretory/Unknown	1 (1.1%)
**ISS stage**—*n* (%)	
I	19 (21.8%)
II	23 (26.4%)
III	45 (51.7%)
**R-ISS stage** (available in 76 patients)—*n* (%)	
I	9 (11.8%)
II	34 (44.7%)
III	33 (43.4%)
**High-risk cytogenetics** (any)—*n*/N (%)	16/73 (21.9%)
del(17p)	2/73 (2.7%)
t(4;14)	4/73 (5.5%)
t(14;16)	4/73 (5.5%)
1q gain/amplification	8/73 (11.0%)
**Extramedullary disease**—*n* (%)	52 (59.8%)
**Triple-class refractory**—*n* (%)	86 (98.9%)
**Penta-class refractory**—*n* (%)	64 (73.6%)
**Line of therapy**—median (IQR; range)	5 (5–6; 2–7)
2–4	8 (9.2%)
5	47 (54.0%)
6	18 (20.7%)
7	14 (16.1%)
**Prior autologous SCT**—*n* (%)	75 (86.2%)
**BM plasma cells ≥ 50%**—*n* (%)	36 (41.4%)
**LDH, U/L**—median (IQR)	219 (177.5–276.5)
LDH > 300 U/L—*n* (%)	19 (21.8%)
**eGFR, mL/min**—median (IQR)	73 (47.1–95.0)
eGFR ≥ 60 mL/min—*n* (%)	57 (65.5%)
**β2-microglobulin, mg/L**—median (IQR)	5.9 (3.3–10.3)
**Albumin, g/L**—median (IQR)	37 (32.1–40.5)
**CRP, mg/L**—median (IQR)	9.3 (3.4–27.8)
**Total IgG, mg/dL**—median (IQR)	603 (385.5–1730)
**Hypogammaglobulinemia** ^a^—*n*/N (%)	23/86 (26.7%)
**Neutrophil count, K/µL**—median (IQR)	2.65 (1.98–3.71)
**Platelet count, K/µL**—median (IQR)	110 (47–159)
**Lymphocyte count, K/µL**—median (IQR)	0.77 (0.45–1.34)
**Prophylactic IVIg**—*n* (%)	68 (78.2%)

^a^ Hypogammaglobulinemia was defined as total IgG level < 400 mg/dL. Abbreviations: IQR, interquartile range; ECOG, Eastern Cooperative Oncology Group; ISS, International Staging System; R-ISS, Revised ISS; SCT, stem cell transplantation; BM, bone marrow; LDH, lactate dehydrogenase; eGFR, estimated glomerular filtration rate; CRP, C-reactive protein; IgG, immunoglobulin G; IVIg, intravenous immunoglobulin; PI, proteasome inhibitor; IMiD, immunomodulatory drug.

**Table 2:** Hematologic Response at 3 and 6 Months.

Response Category	3-Month (*n* = 87)	6-Month (*n* = 87)
**Not assessed**	38 (43.7%)	54 (62.1%)
Death before landmark response assessment	13	21
Progression before landmark	5	7
Insufficient follow-up	12	23
Missing assessment	8	3
**Documented sCR** ^a^	0 (0.0%)	0 (0.0%)
**CR**	15 (17.2%)	21 (24.1%)
**VGPR**	13 (14.9%)	5 (5.7%)
**PR**	13 (14.9%)	1 (1.1%)
**SD**	3 (3.4%)	1 (1.1%)
**PD**	5 (5.7%)	5 (5.7%)
**ORR (≥PR), evaluable** (95% CI)	41/49 (83.7%; 70.3–92.7)	27/33 (81.8%; 64.5–93.0)
**ORR (≥PR), ITT** (95% CI)	41/87 (47.1%; 36.3–58.1)	27/87 (31.0%; 21.5–41.9)
**≥VGPR, evaluable** (95% CI)	28/49 (57.1%; 42.2–71.2)	26/33 (78.8%; 61.1–91.0)
**≥CR, evaluable** (95% CI)	15/49 (30.6%; 18.3–45.4)	21/33 (63.6%; 45.1–79.6)
**ORR (≥PR), Mitt** (95% CI)	41/75 (54.7%; 42.7–66.2)	27/64 (42.2%; 29.9–55.2)

^a^ sCR criteria were not uniformly assessed across centers and timepoints; therefore, “0 (0.0%)” indicates no documented sCR rather than a fully assessed endpoint. PET-CT assessment was available in 35 patients (40.2%) at 3 months and 23 patients (26.4%) at 6 months. Metabolic response (CMR + PMR) was observed in 88.6% of patients at 3 months and 69.6% at 6 months. At 3 months, CMR, PMR, SMD, and PMD rates were 57.1%, 31.4%, 0%, and 11.4%, respectively. At 6 months, corresponding rates were 34.8%, 34.8%, 4.3%, and 26.1%.

**Table 3:** Univariate Cox Proportional Hazards Analysis for PFS and OS.

Variable (vs. Reference)	PFS	*p*	OS	*p*
	HR (95% CI)		HR (95% CI)	
ECOG PS ≥ 2 (vs. 0–1)	2.00 (1.05–3.83)	**0.036**	2.19 (1.04–4.63)	**0.040**
Prior SCT (vs. no SCT)	0.33 (0.13–0.84)	**0.021**	0.21 (0.08–0.60)	**0.003**
LDH > 300 U/L (vs. ≤300)	3.01 (1.52–5.96)	**0.002**	4.84 (2.27–10.31)	**<0.001**
eGFR ≥ 60 mL/min (vs. <60)	0.50 (0.26–0.95)	**0.035**	0.43 (0.21–0.89)	**0.024**

**Table 4:** Multivariable Cox Proportional Hazards Analysis (Final 3-Variable Models).

**Variable**	**PFS (37 Events)**	***p***	**OS (29 Events)**	***p***
	**aHR (95% CI)**		**aHR (95% CI)**	
**ECOG PS ≥ 2 (vs. 0–1)**	1.87 (0.97–3.63)	0.063	2.04 (0.95–4.40)	0.068
**LDH per 100 U/L**	1.75 (1.36–2.25)	**<0.001**	1.94 (1.50–2.51)	**<0.001**
**Line of therapy (per additional prior line)**	1.22 (0.86–1.72)	0.267	1.35 (0.91–2.01)	0.134

**Table 5:** Safety and Treatment Modifications (*n* = 87).

Adverse Event	*n* (%)
**CRS (any grade)**	60 (69.0)
Grade ≥ 3 CRS	5 (5.7)
Median onset, day (IQR)	1 (1–2)
Tocilizumab use	22 (25.3)
**ICANS (any grade)**	4 (4.6)
Grade ≥ 3 ICANS	0 (0.0)
**Grade ≥ 3 hematologic toxicity**	
Neutropenia	21 (24.1)
Thrombocytopenia	34 (39.1)
Lymphopenia	65 (74.7)
Tumor lysis syndrome	1 (1.1)
**Treatment modifications**	
Treatment interruption	28 (32.2)
Permanent discontinuation	22 (25.3)

**Table 6:** Infection Outcomes (*n*= 87).

Outcome	*n* (%)
Any documented infection	52 (59.8%)
Grade 1–2 infection	47 (54.0%)
Grade ≥ 3 infection	34 (39.1%)
**Site of grade ≥ 3 infections**	
Pneumonia	21 (24.1%)
Gastrointestinal	6 (6.9%)
Skin/soft tissue	5 (5.7%)
Urinary tract	2 (2.3%)
Febrile neutropenia	22 (25.3%)
Sepsis	16 (18.4%)
CMV event requiring antiviral therapy	21 (24.1%)
HBV reactivation (among HBsAg-positive and/or anti-HBc-positive patients, *n* = 18)	0 (0.0%)

## Data Availability

The datasets generated and/or analyzed during the current study are available from the corresponding author on reasonable request.

## Supplementary Materials

The following supporting information can be downloaded at: https://www.mdpi.com/article/10.3390/jcm15166232/s1.
