# Supplementary material for: Elranatamab in Relapsed/Refractory Multiple Myeloma: A Multicenter Real-World Study from Türkiye
Source: J Clin Med. 2026 Aug 12;15(16):6232. doi: 10.3390/jcm15166232 (PMC13513165; doi:10.3390/jcm15166232)
Supplement: Supplementary file 1 [file jcm-15-06232-s001.zip › jcm-4440713-supplementary.pdf]

SUPPLEMENTARY MATERIAL

Table S1. Numbers at Risk, Interval Events, and Kaplan–Meier Estimates for Overall Survival and Progression-Free Survival.

Time (months)	OS: N at risk	OS interval events	OS rate (%)[a]	PFS: N at risk	PFS interval events	PFS rate (%)[a]
0	87	---	100.0	87	---	100.0
3	52	19	75.9	49	24	70.3
6	31	3	70.2	29	6	60.0
9	11	5	53.0	9	6	43.2
12	2	2	37.1*	2	1	38.4*

*[a]Rates are Kaplan--Meier estimates (accounting for censoring) at each timepoint. Interval events represent the number of events that occurred during the interval from the previous timepoint to the current timepoint (e.g., 19 OS events occurred between months 0 and 3; 3 OS events occurred between months 3 and 6). The total cumulative OS events at 12 months is 19 + 3 + 5 + 2 = 29, consistent with the reported total of 29 deaths. N at risk excludes patients who were censored or experienced an event before each timepoint. *12-month estimates are preliminary and should be interpreted with caution due to limited follow-up maturity (median 6.2 months; only 2 patients at risk).*

Table S2. Exploratory IVIg and Infection Analysis (Unadjusted).

**Outcome**	**IVIg (n = 68)**	**No IVIg (n = 19)**
Hospitalization-requiring infection	32 (47.1%)	2 (10.5%)

Note: This exploratory, unadjusted analysis should be interpreted cautiously because IVIg initiation dates were unavailable and treatment allocation may have been influenced by underlying infection risk. Consequently, it was not possible to determine whether IVIg exposure preceded hospitalization-requiring infection. Accordingly, the findings are presented descriptively and are not intended to establish a causal effect of IVIg on infection outcomes or to suggest either benefit or harm associated with IVIg use.

Table S3. CMV PCR Surveillance and Antiviral Treatment.

CMV-related variable	n/N (%)	CMV events, n/N (%)
**PCR monitoring frequency**		
No routine PCR surveillance	19/87 (21.8%)	1/19 (5.3%)
Only when clinically suspected	27/87 (31.0%)	6/27 (22.2%)
Monthly	2/87 (2.3%)	0/2 (0.0%)
Every 2 weeks or more frequently	39/87 (44.8%)	14/39 (35.9%)
**Antiviral agents among treated CMV events (n = 21)**		
Ganciclovir	6/21 (28.6%)	---
Valganciclovir	17/21 (81.0%)	---
Foscarnet	0/21 (0.0%)	---

Note: Antiviral-agent categories are not mutually exclusive because 2 patients received both ganciclovir and valganciclovir; 4 received ganciclovir alone and 15 received valganciclovir alone. Differences in CMV event detection across surveillance groups are descriptive and may reflect ascertainment bias and center-level practice variation.
